# Impact of the use of cryobank samples in a selected cattle breed: a simulation study

**DOI:** 10.1186/1297-9686-43-36

**Published:** 2011-11-02

**Authors:** Grégoire Leroy, Coralie Danchin-Burge, Etienne Verrier

**Affiliations:** 1 AgroParisTech, UMR 1313 Génétique Animale et Biologie Intégrative, 16 rue Claude Bernard, 75231 Paris 05, France; 2 INRA, UMR 1313 Génétique Animale et Biologie Intégrative, 78350 Jouy-en-Josas, France; 3 Institut de l'Elevage, 149 rue de Bercy, 75012 Paris, France

## Abstract

**Background:**

High selection pressure on domestic cattle has led to an undesirable increase in inbreeding, as well as to the deterioration of some functional traits which are indirectly selected. Semen stored in a cryobank may be a useful way to redirect selection or limit the loss of genetic diversity in a selected breed. The purpose of this study was to analyse the efficiency of current cryobank sampling methods, by investigating the benefits of using cryopreserved semen in a selection scheme several generations after the semen was collected.

**Methods:**

The theoretical impact of using cryopreserved semen in a selection scheme of a dairy cattle breed was investigated by simulating various scenarios involving two negatively correlated traits and a change in genetic variability of the breed.

**Results:**

Our results indicate that using cryopreserved semen to redirect selection will have an impact on negatively selected traits only if it is combined with major changes in selection objectives or practices. If the purpose is to increase genetic diversity in the breed, it can be a viable option.

**Conclusions:**

Using cryopreserved semen to redirect selection or to improve genetic diversity should be carried out with caution, by considering the pros and cons of prospective changes in genetic diversity and the value of the selected traits. However, the use of genomic information should lead to more interesting perspectives to choose which animals to store in a cryobank and to increase the value of cryobank collections for selected breeds.

## Background

Within the context of farm animal biotechnologies, cryopreservation is one of the most useful tools for selection improvement, dissemination of genetic progress and *ex situ *conservation. In its Global Plan of Action, the FAO [1] recommended the implementation of *ex situ *programmes to complement *in situ *conservation of animal genetic resources. It was also suggested that cryopreserved bio-specimens could be used as a backup material to redirect the selection scheme of a given breed, if needed [2,3]. Consequently, several gene banks have been created with different strategies and policies that vary with the breed, species, and country concerned [4,5] and methods have been proposed to use *ex situ *genetic resources to optimise the management of genetic diversity in endangered breeds [6]. Breeds with large populations are subject to high selection pressures and have rates of inbreeding greater than the desired values [7]. In these cases, the use of stored semen from male ancestors has seldom been investigated, although breeding organisations could be interested in doing so. For instance, in the dairy cattle breed Abondance (a local selected breed in the French Northern Alps), the semen of a bull born in 1977 (called Naif), which was rarely used in the 1980's, was used from 2004 to 2007, to produce 20 young bulls in order to reintroduce some genetic variability in the breed.

Depending on the country, different strategies have been implemented to sample individuals for national collections. In the Netherlands, most of the tested bulls are sampled for preservation in the gene bank [8], while in the USA, the selection of animals for cryopreservation is aimed at optimizing genetic diversity within the collection, by sampling animals from clusters determined through computed genealogical relationships [9]. In France, based on the idea that individuals sampled for a cryobank should be as diverse as possible and carry special genotypes [10], regulations have been implemented to conserve frozen sperm from three main origins: (I) animals from endangered breeds, (II) original animals from non-endangered breeds (with either extreme positive or negative Estimated Breeding Values (EBV), carrying rare alleles or representing rare pedigree lines), and (III) representative animals from non-endangered breeds [2].

The purpose of this study was to analyse the efficiency of current cryobank sampling methods by investigating the benefits of using cryopreserved semen in a selection scheme several generations after the semen was collected. Based on simulations, we examined two situations in which cryopreserved sperm was used (1) to redirect the selection goal, by including a trait which, in the past, had shown a negative correlated selection response (e.g. fertility in dairy cattle), and (2) to limit the loss of genetic diversity in the breed. The impact of using cryopreserved sperm was measured by estimating the evolution of two negatively correlated traits and the evolution of the breed's genetic diversity, assessed through pedigree information.

## Methods

### Simulated breed

A simplified cattle breed was simulated with 13 discrete generations, each consisting of 100 males and 10000 females. In each generation, 10 bulls and 50 cows were chosen as parents of the male progeny, and 20 bulls and 10000 cows were chosen as parents of the female progeny (with no selection on the dam to dam path). Mating was random resulting in random variation of progeny size among parents, i.e. the sire and dam of a given newborn were randomly chosen in the corresponding lists of parents.

### Simulation of genetic values and EBV

We considered two traits A and B. Trait A corresponded to a production trait which had been recently and intensively selected and improved (such as milk production in dairy cattle). Trait B corresponded to a functional trait which had deteriorated because of a negative correlation with trait A (e.g. fertility or longevity). The genetic standard deviation of each trait (σ*_A _*and σ*_B_*, respectively) was set to 1 and the correlation between traits (ρ) was set to -0.3.

For each trait, an additive polygenic model was assumed and the simulation of correlated genetic values was based on the bivariate normal distribution (see, e.g. [11]). At generation 0 (base population), genetic values for trait A were randomly and independently drawn from a *N *(0, 1) distribution. For a given individual (*i*), the genetic value for trait B (*B_i_*) was generated from its value for trait A (*A_i_*):

(1) $$B_{i}=\rho \;A_{i}+\surd \left(1-\rho^{2}\right)\beta_{i}$$

where β*_i _*is a *N *(0, 1) random number independent of *A_i_*.

In the following generations, genetic values of individual *i *were simulated from the genetic values of its sire (*A_p _*and *B_p_*) and its dam (*A_m _*and *B_m_*), taking into account the parent's coefficients of inbreeding (*F_p _*and *F_m_*, resp.) [12,13]:

(2.a) $$A_{i}=1\;/\;2\left(A_{p}+A_{m}\right)+\gamma_{i}\surd 1\;/\;2\left[1-\left(F_{p}+F_{m}\right)∕2\right]$$

(2.b) $$B_{i}=1\;/\;2\left(B_{p}+B_{m}\right)+\delta_{i}\surd 1\;/\;2\left[1-\left(F_{p}+F_{m}\right)∕2\right]$$

In these equations, γ*_i _*and δ*_i _*are two numbers randomly drawn from a *N *(0, 1) bivariate normal distribution with a correlation equal to ρ.

EBV were directly simulated from genetic values, assuming an evaluation procedure leading to an accuracy (*CD *= square of the correlation between the EBV and the true genetic value) equal to 0.6 for bulls and 0.4 for cows, whatever the trait and the generation considered. Therefore, the EBV of a given individual for trait A (*EBVA_i_*) and for trait B (*EBVB_i_*) were computed as follows:

(3.a) $$EBVA_{i}=CD_{i}A_{i}+\varepsilon_{i}\surd CD_{i}\left(1-CD_{i}\right)$$

(3.b) $$EBVB_{i}=CD_{i}B_{i}+\phi_{i}\surd CD_{i}\left(1-CD_{i}\right)$$

where ε*_i _*and ϕ*_i _*are two independent numbers drawn from a *N*(0, 1) distribution. Finally, a Total Merit Index (*TMI_i_*) was computed, weighting the two EBV by *w_A _*and *w_B _*= 1 - *w_A_*, respectively:

(3.c) $$TMI_{i}=w_{A}EBVA_{i}+w_{B}EBVB_{i}$$

### Sampling and use of cryopreserved semen

Simulations comprised two stages. During stage 1 (generations 0 to 8), the lists of parents were selected based on their EBV for trait A only, without considering the evolution of the genetic mean for trait B or the average coefficient of inbreeding. During stage 2 (generations 9 to 12), the bulls were also used to improve trait B or to introduce genetic diversity in the breed.

During stage 1, the semen of some bulls was sampled and cryopreserved if the animals fulfilled one of the three following conditions, which correspond to the current sampling rules of the French National Cryobank for type "II" (original bulls) [2]:

- (i) *EBVA *is three standard deviations above or below the mean of the generation,

- (ii) *EBVB *is two standard deviations above the mean of the generation (trait B is considered as a functional trait and for functional traits, only animals above the average are considered),

- (iii) the bull is a sire of sires with no male offspring selected after the evaluation process (these bulls were actually selected with one generation lag).

To check the validity of this elaborate sampling method, we tested a simpler sampling method (similar to the one used in the Netherlands), where the semen of all young bulls is stored in the cryobank.

In the simulations performed here, we investigated the impact of a one-time use (i.e. during a single generation) of cryopreserved semen.

At generation 9, four bulls with cryopreserved semen were selected (hereafter referred to as 'cryobank bulls'), these bulls fulfilling one of the following conditions either (i) they are the best cryobank bulls for the *TMI_i _*or (ii) they have the lowest average kinship with the existing population (males and females taken together). We studied the impact of various selection orientations (use of cryopreserved semen, conservation of male lines, etc.) only on the male path, because applying the above conditions on the female path would be much more restrictive, less effective, and would require a larger amount of semen, all the more since the number of doses is generally limited in cryobanks (200, in France) [2].

For these reasons, we considered that cryobank bulls were used only to procreate young bulls for progeny testing. The 9^th ^generation of young bulls was then generated using either the bulls from the cryobank or the group of 10 sires selected as described in previous sections. Depending on the scenario (see following section), 0, 40 or 80 individuals (among the 100 newborn bull calves) were sired randomly by one of the four selected cryobank bulls.

### Simulation scenarios and results

Six simulation scenarios were completed with two main options (Table 1).

**Table 1:** Description of simulation scenarios

Scenarios		Use of cryobank bulls in generation 9		Selection scheme during stage 2 (generations 9-12)	
Objective	Code	Selection of cryobank bulls	% of male offspring	Selection criterion	Use of sire of bulls

Improving trait B	b1	Higher *TMI *value	40	*EBVA*	No change
	b2	Not used	0	*TMI*	No change
	b3	Higher *TMI *value	40	*TMI*	No change

Maintaining genetic diversity	d1	Minimizing kinship with current population	40	*EBVA*	No change
	d2	Not used	0	*EBVA*	Conservation of male lines
	d3	Minimizing kinship with current population	40	*EBVA*	Conservation of male lines

*EBVA*: estimated breeding value for trait A; *EBVB*: estimated breeding value for trait B; *TMI*: total merit index computed as the weighted sum of *EBVA *and *EBVB*

Firstly, in scenario "b", emphasis was put on the selection of both traits B and A. To achieve this goal, three methods were compared:

- b1: at generation 9, the four bulls with the highest *TMI *(*w_B _*= 0.5) were used to sire 40% of the young bulls, while the selection criterion during stage 2 remained unchanged (improving *EBVA*). The other young bulls were sired by bulls randomly sampled within the group of 10 sires;

- b2: at generation 9, no cryobank bull was used, and during stage 2, *TMI *(*w_B _*= 0.5) was used as the selection criterion instead of *EBVA*;

- b3: at generation 9, the four cryobank bulls with the highest *TMI *were used to sire 40% of the young bulls, and during stage 2, *TMI *was used as the selection criterion instead of *EBVA*. To test more or less drastic selection changes, scenario b3 was tested with an increasing weight given to trait B (*w_B _*increasing from 0.5 to 1).

Secondly, in scenarios "d", emphasis was put on genetic variability while trait A remained the breeding goal. Three methods were also compared:

- d1: at generation 9, the four cryobank bulls having the lowest kinship with the existing population (scenario b1) were used to sire 40% of the young bulls;

- d2: at generation 9, no cryobank bull was used, while during stage 2, the progenies on the sire to sire path were given the same size i.e. for each sire of sires, 10 male offspring were created among which those with the two best *EBVA *became the sires of dams and that with the best *EBVA *became a sire of sires;

- d3: at generation 9, the four cryobank bulls having the lowest kinship with the existing population (scenario b1) were used to sire 40% of the young bulls, while during stage 2, selection was used to equalise progeny sizes on the sire to sire path.

Simulations were performed with 1000 runs for each scenario. For each generation, individual inbreeding coefficients and genetic values were computed and averaged for the entire male and female populations. The individual coefficients of kinship were also computed and averaged over males only and over the entire populations. The proportion of genes originating from cryobank bulls was computed on the basis of the gene dropping procedure (one locus averaged over the 1000 runs).

## Results

### Stage 1: evolution of selected traits, diversity loss, and sampling of cryobank bulls

**Figure 1:**
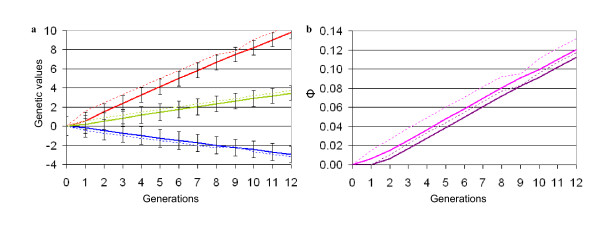
**Changes in genetic values (a) and in genetic diversity (b) (scenario b1)**. Dotted lines: young bulls; solid lines: whole population; red: trait A, blue: trait B; green: average between A and B; purple: inbreeding F; pink: kinship *Φ*.

As expected, the results of the different scenarios did not differ significantly for generations 0 to 8 given that in stage 1, the conditions were the same whatever the option chosen, (here we present results averaged over the 1000 runs of one scenario only). With the parameters chosen for the simulation, each sire of sires had on average 10 male offspring (across sires standard deviation s.d. = 2.9) and each sire of dams had on average 500 female offspring (across sires s.d. = 21.6). As expected (see Figure 1), selection on trait A during stage 1 led to a major increase in the mean of this trait (+ 6.7 initial genetic standard deviation) from generation 0 to 8, while at the same time, the mean of B decreased to a lesser extent (-2 initial genetic standard deviation). The average coefficient of inbreeding increased simultaneously. Young bulls were slightly more inbred than cows, as they originated from a smaller number of sires and dams. In parallel (generation 0 to 8), the average coefficient of kinship among the young bulls and among the entire population increased to 8.1% and 6.9%, respectively.

An average of 31 cryobank bulls was sampled per replicate, 58% being sampled because of outstanding *EBVB *(see Table 2). Table 2 shows that cryobank bulls chosen for their genetic diversity were generally born earlier than others, which can be explained by the fact that they were chosen with one generation lag compared to other sampling procedures.

**Table 2:** Average number and birth generation of bulls selected for conservation

Cryobank sampling criterion	Number of bulls per replicate	% of all cryobank bulls	Birth generation of cryobank bulls
> + 3 s.d. *EBVA*	0.92 *[0.96]*	3%	3.52 *[1.96]*
< - 3 s.d. *EBVA*	0.95 *[0.94]*	3%	3.56 *[2.03]*
> + 2 s.d. *EBVB*	18 *[3.22]*	58%	3.5 *[0.45]*
Sire of sires with no male offspring selected	11.2 *[2.48]*	36%	2.96 *[0.48]*

Total	30.9	_	3.3

In brackets, are given the standard deviations (s.d.) between replicates; note that the sum of the cryobank bulls sampled in each category is larger than the total number of cryobank bulls because some bulls were chosen for several criteria at the same time

### Stage 2 in scenarios b: change in breeding goals

As shown in Figure 1, introducing cryobank bulls with exceptional *TMI *without changing the selection criterion during stage 2 (scenario b1) had a temporary impact on traits A and B as well as on the diversity indicators of the young bulls. At the whole population level, the impact was negligible, since young bulls sired by cryobank bulls were rarely subsequently selected as sires: three generations after introduction (generation 12), the cryobank contribution to genetic diversity was less than 3% (Table 3).

**Table 3:** Origin and impact of cryobank bulls used in the different scenarios

		Scenario*			
		
		b1	b3	d1	d3
Proportion of bulls used according to the sampling criterion (%)	> +3 s.d. *EBVA*	3.3 *[8.7]*	3 *[8.8]*	1 *[1.8]*	1 *[1.6]*
	< -3 s.d. *EBVA*	3.1 *[8.8]*	3 *[8.2]*	4 *[1.0]*	4 *[1.0]*
	> +2 s.d. *EBVB*	67.8 *[21.2]*	67 *[21.6]*	72 *[21.7]*	71 *[20.6]*
	Sire of sires with no male offspring selected	26.3 *[21.2]*	27 *[21.6]*	24 *[20.7]*	25 *[20.2]*

Birth generation of cryobank bulls used		6.6 *[0.3]*	6.6 *[0.3]*	0.3 *[0.3]*	0.3 *[0.3]*

Proportion of genes originating from cryobank bulls (%)	Generation 10	3.6 *[2.3]*	7.3 *[2.8]*	0. *[0.1]*	8.8 *[0.9]*
	Generation 12	2.8 *[2.9]*	6.5 *[4.2]*	0. *[0.0]*	6.8 *[2.3]*

In this case, trait B accounts for 50% of the total merit index

*in scenarios b2 and d2, no cryobank bull was used; in brackets are given the standard deviations (s.d.) between replicates

When *TMI *was used as a selection criterion (considering *w_B _*= 0.5), without using cryobank bulls (scenario b2), there was a per generation increase in the mean of trait B from generation 9 on (b1: -0.3 vs b2: +0.4), while the genetic gain for trait A decreased (b1: +1.0 vs b2: +0.4, see additional file 1). The change in breeding goals had no impact on diversity indicators.

Combining the use of cryobank bulls and *TMI *as a selection criterion (scenario b3 for *w_B _*= 0.5) resulted in a slight but significant (*P *< 0.001) reduction in average kinship (-0.3% between scenario b2 and b3, with 40% of the males from generation 9 sired by cryobank bulls, see additional file 2). Concerning the selected traits, the genetic gain for trait A decreased slightly when cryobank bulls were used (-0.12 between scenarios b2 and b3, *P *< 0.001), while the genetic gain for trait B increased slightly (+0.06 between scenarios b2 and b3, *P *= 0.02). These tendencies increased slightly when 80% of the males from generation 9 were sired by cryobank bulls (see additional file 2). According to the results from Table 3, cryobank bulls contributed to 6.5% of the diversity three generations after their introduction. It should be noted that the cryobank bulls used were generally sampled in recent generations, their average birth generation being 6.6 (Table 3).

**Figure 2:**
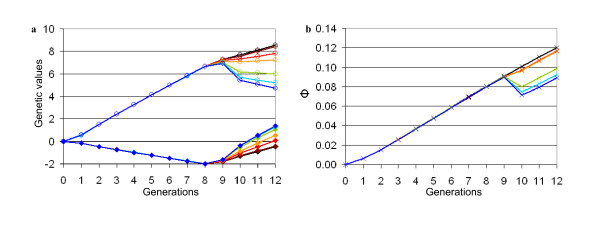
**Changes in genetic values (a) and in average kinship (b), when trait B was added to selection goals**. Scenario b3 and whole population are considered with the weight *w_B _*of trait B increasing for computation of the total merit index. Black: *w_B _*= 0 (scenario *b1*); brown: *w_B _*= 0.5; red: *w_B _*= 0.6; orange: *w_B _*= 0.7; green: *w_B _*= 0.8; light blue: *w_B _*= 0.9; dark blue: *w_B _*= 1; o: genetic value for trait A; ♦: genetic value for trait B; x: kinship *Φ*.

As a result of the increased weight of trait B within *TMI *(see Figure 2), there was a per generation increase in genetic gain for trait B, while there was a slightly lower increase or even a decrease in genetic gain for trait A, as well as in average kinship, when trait B accounted for more than 80% of EBV. When only trait B was taken into account for *TMI*, the genetic value of traits A and B reached 4.7 and 1.37, respectively at generation 12 (versus 8.4 and -0.41 respectively when *w_B _*= 0.5), while average kinship reached 8.9% at generation 12 (versus 11.9% when *w_B _*= 0.5).

### Stage 2 in scenarios d: improvement in genetic diversity

**Figure 3:**
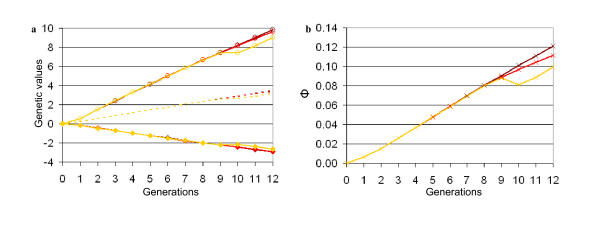
**Changes in genetic values (a) and in average kinship (b), when the aim was to manage genetic diversity**. The whole population is considered Brown: no change in selection; cryobank bulls used to produce 40% of male offspring (scenario d1); red: conservation of male lines (scenario d2) (curve overlapping the preceding one); yellow: conservation of male lines and cryobank bulls used to produce 40% of male offspring (scenario d3); o: genetic value for trait A; ♦: genetic value for trait B; dotted line: average genetic value between A and B; x: kinship *Φ*.

As shown in Figure 3, the use of cryobank bulls with a minimised kinship with the current generation (scenario d1), had no impact if the selection policy was not modified, since none of the offspring of the cryobank bulls were selected as sires. Equalising progeny sizes on the sire to sire path alone (scenario d2) decreased diversity a little less (in generation 12, *Φ *= 12% for scenario d1 and *Φ *= 11% for scenario d2), with an almost negligible impact on genetic progress. Combining this option with the introgression of cryobank bulls (scenario d3) resulted in a significant reduction in average kinship (-2% in comparison to d1). Under such a scenario, the genetic mean of trait B also increased slightly (+0.3 between scenario d1 and d3, *P *< 0.001), while that of trait A and the average of both traits decreased slightly (-0.08 and -0.02 respectively, between scenarios d1 and d3, *P *< 0.001). It should be noted that most of the cryobank bulls used originated from the founder population, their average birth generation being 0.3 (Table 3).

Modifying which bulls entered the cryobank by preserving semen for all the young bulls did not significantly alter the results of scenarios b3 and d3, either for the selected traits or for kinship evolution (data not shown). It should be noted that in this case, the average birth generation of the cryobank bulls used was 7, in scenario b3 (instead of 6.6, in the first cryobank sampling method), and 0, in scenario d3 (instead of 0.3, in the first cryobank sampling method).

## Discussion

In this study, we assessed the impacts of using cryopreserved bull semen either to redirect selection or to improve the genetic variability of a selected cattle breed. Simulation parameters were chosen as a compromise between realism in the scenarios, their applicability, and the simplicity of the model. For instance, with respect to the choice of population size, a breed with 20 breeding males and 10000 potential dams could be considered quite small, especially with reference to the FAO endangerment status [14]. In our simulation, sires and dams were randomly chosen from lists of reproducers. This differs significantly from what occurs in real breeds, in which an unbalanced use of reproducers is frequently the case, leading to a reduced size of the effective population. In terms of effective size, our breed would correspond to a much larger population with a similar inbreeding rate per generation (1.07%) to that found in real dairy cattle breeds e.g. [15].

Concerning sampling conditions in the simulations, as mentioned above, the procedure chosen to select bulls for cryopreservation is similar to that currently applied in France. This choice was made to test if bulls selected this way could be effectively used in a selected breed. Compared to the case in which all young bulls are sampled for cryopreservation (which corresponds more or less to the current procedure in the Netherlands), the results were basically the same. This shows that the French sampling procedure is reasonably efficient to select useful bulls, and could be applied in situations when only a limited number of semen samples can be stored in a cryobank (for financial reasons, for instance).

One of the main conclusions of this study is that using cryopreserved semen is relevant for a breed for which major changes in selection objectives or practices are considered. Since genetic progress is rapid in dairy cattle breeds (e.g. [16]), a bull for which semen has been stored for a few generations, is likely to have a lower genetic value than current bulls, if the selection goals remain the same. Thus the latter's offspring may not be used, as illustrated by scenarios b1 and d1, and using cryobank bulls is then meaningless. The results of scenario b3 demonstrate that using cryobank bulls has a significant impact on the selected traits and on genetic diversity only if a relatively large change is implemented in the selection programme (i.e. introducing a new trait formerly negatively selected but subsequently accounting for more than 50% of EBV). Under that scenario, when trait B accounted for less than 70% of EBV, the cryobank bulls selected were those more recently collected, since they generally had a higher value for trait A than older cryobank bulls, which compensated for a slightly lower value for trait B. When trait B accounted for 80% or more of EBV, most of the cryobank bulls finally used, originated from generation 0 (data not shown), which explains the sudden decrease in average kinship after introgression of the cryobank bulls (see Figure 2). Therefore if managers of a selection scheme want to redirect breeding goals, using cryobank bulls is viable only if the breeding goals are subjected to a major modification (i.e. if the weight of the new trait accounts for more than 50% of EBV). Our results also indicate that cryobank bulls that have been sampled for functional traits with high EBV will tend to be used more frequently than other cryobank bulls, independently of the aim.

If the objective is to introduce genetic diversity into the breed, using cryobank bulls appears to be a valid choice. However, it is imperative that other measures are also taken to guaranty that genes are spread within the breed i.e. either conserving male lines (scenario d3), when their use is promoted among breeders, or setting up more restrictive and effective breeding schemes. Several methods of varying complexity have been proposed to minimise kinship [6], or to maximise breeding values for a predefined inbreeding rate [17], or to minimise average kinship for a desired average EBV [18], usually by optimising the contribution of reproducers.

On the one hand, decreasing inbreeding in a selected breed may improve selected traits; for instance, it has been shown that in Holstein cattle, milk production (over 305 days) can decrease by about 20 litres per 1% inbreeding increase [19]. On the other hand, using semen from cryobank bulls has a negative impact on previously selected traits, as illustrated by our simulations. In the case of local breeds, in which genetic progress is not as effective as in breeds with a larger population size, the difference in EBV between current bulls and bulls from earlier generations should be minimised. This could lead to an effective use of cryobank bulls to reintroduce diversity without overly affecting selected traits. As an illustration in the Abondance breed, one of the male offspring of the bull born in 1977 was found to have quite a high EBV (Vaccin, born in 2003, [20]), and was therefore recently confirmed as a sire of dams. Among all the sires of dams, this bull shared the lowest average kinship with the 2004-2007 female cohorts (4.6% vs. 6.5% on average, personal communication). The impact of using this bull on the genetic variability of the breed remains to be assessed.

## Conclusions

Based on our results, using semen from cryobank bulls should be useful either to introduce drastic changes in selection goals or to reintroduce genetic diversity within a given population. However, it is important to carefully assess the pros and cons of the potential changes in genetic diversity and values of the selected traits.

Our simulations were based on a classic quantitative selection scheme. Recent progress in genomic tools should make it possible to identify semen from cryobank bulls that share specific alleles or QTL of interest for selection. This could then be taken into account when choosing cryobank bulls as well as how they will be used. Using such reproducers should be investigated in further studies, which opens exciting perspectives for an improved exploitation of cryobank collections.

## Competing interests

The authors declare that they have no competing interests.

## Authors' contributions

EV, CDB and GL jointly conceived the design of the study and discussed the results. GL wrote and checked the simulation program. GL wrote the first draft of the manuscript, which was then modified by CDB and EV. All authors read and approved the final manuscript.

## Supplementary Material

Additional file 1 **Changes in genetic values (a) and in genetic diversity (b) (scenario b2)**. The data represent the simulation results for scenario b2. Dotted lines: young bulls; solid lines: whole population; red: trait A; blue: trait B; green: average between A and B; purple: inbreeding F; pink: kinship *Φ*. Click here for file

Additional file 2 **Changes in genetic values (a) and in average kinship (b) when trait B was added to selection goals**. The data represent the simulation results when selection is redirected with a new trait accounting for 50% of the total merit index and when the use of semen from cryobank bulls is increased. Scenario b3 and whole population are considered with the weight *w_B _*given to trait B accounting for 50% of the total merit index and an increased use of the semen from cryobank bulls. Brown: no cryobank bull is used (scenario b2); red: cryobank bulls are used to produce 40% of sons (scenario b3); yellow: cryobank bulls are used to produce 80% of sons; o: genetic value for trait A; ♦: genetic value for trait B; dotted line: average genetic value between A and B; x: kinship *Φ*. Click here for file
