# Supplementary figures and images for: Impact of the use of cryobank samples in a selected cattle breed: a simulation study
Source: Genet Sel Evol. 2011 Nov 2;43(1):36. doi: 10.1186/1297-9686-43-36 (PMC3220632; doi:10.1186/1297-9686-43-36)

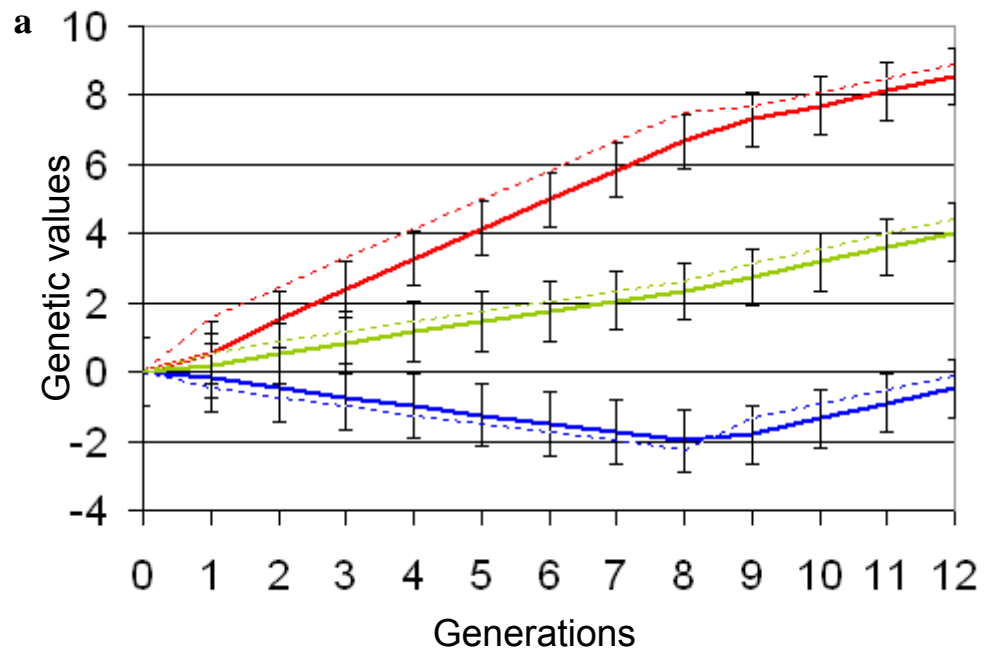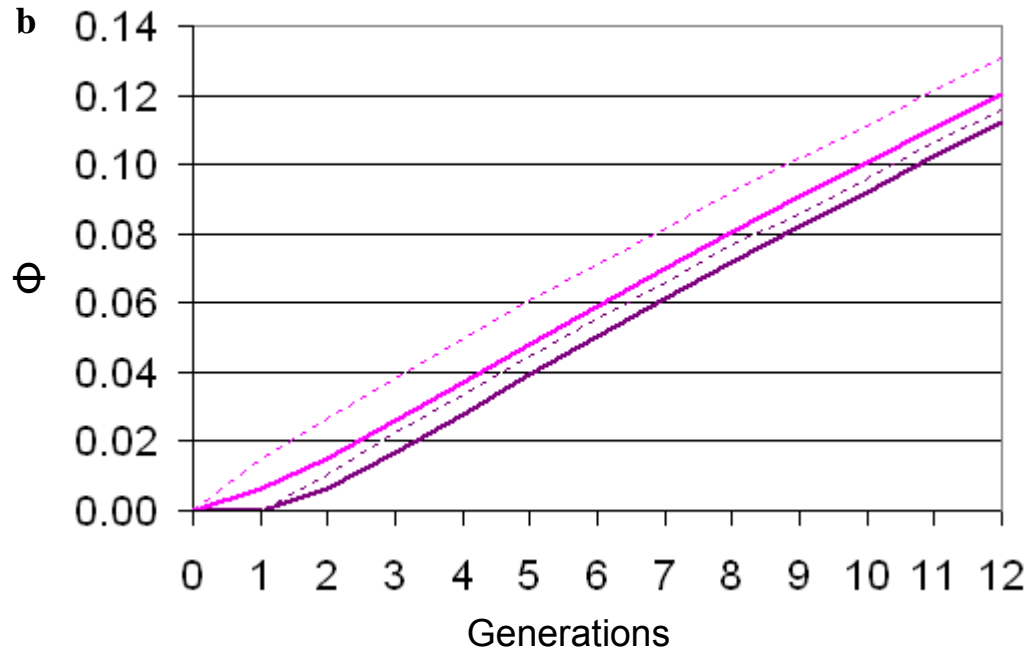

Supplement: Additional file 1 — Changes in genetic values (a) and in genetic diversity (b) (scenario b2). The data represent the simulation results for scenario b2. Dotted lines: young bulls; solid lines: whole population; red: trait A; blue: trait B; green: average between A and B; purple: inbreeding F; pink: kinship Φ. [file 1297-9686-43-36-S1.PDF]

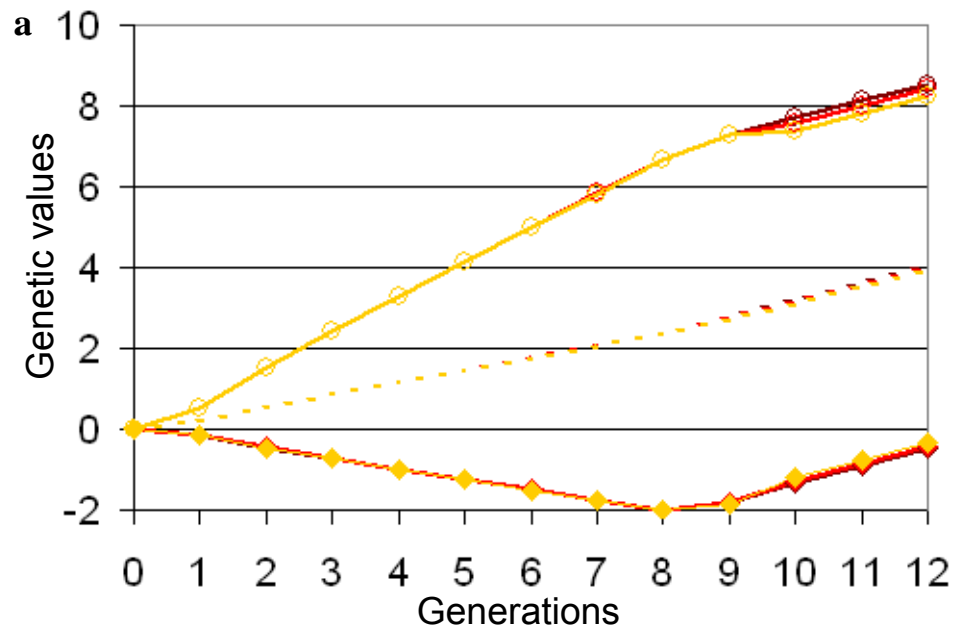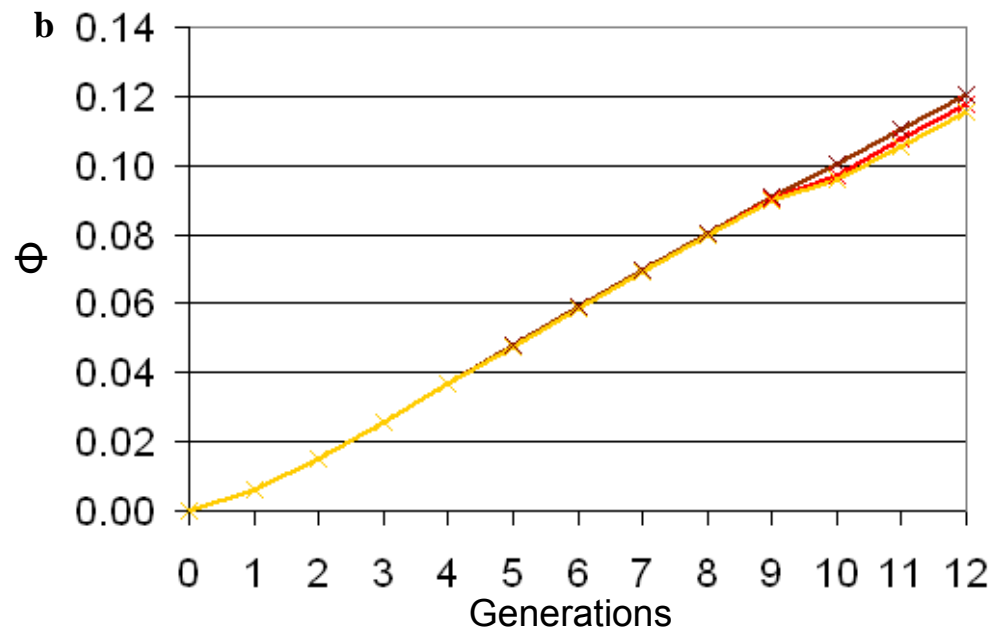

Supplement: Additional file 2 — Changes in genetic values (a) and in average kinship (b) when trait B was added to selection goals. The data represent the simulation results when selection is redirected with a new trait accounting for 50% of the total merit index and when the use of semen from cryobank bulls is increased. Scenario b3 and whole population are considered with the weight wB given to trait B accounting for 50% of the total merit index and an increased use of the semen from cryobank bulls. Brown: no cryobank bull is used (scenario b2); red: cryobank bulls are used to produce 40% of sons (scenario b3); yellow: cryobank bulls are used to produce 80% of sons; o: genetic value for trait A; ♦: genetic value for trait B; dotted line: average genetic value between A and B; x: kinship Φ. [file 1297-9686-43-36-S2.PDF]
